# Working Dogs Drinking a Nutrient-Enriched Water Maintain Cooler Body Temperature and Improved Pulse Rate Recovery After Exercise

**DOI:** 10.3389/fvets.2018.00202

**Published:** 2018-08-28

**Authors:** Brian M. Zanghi, Patrick J. Robbins, Meghan T. Ramos, Cynthia M. Otto

**Affiliations:** ^1^Nestlé Purina Research, St. Louis, MO, United States; ^2^Penn Vet Working Dog Center, University of Pennsylvania School of Veterinary Medicine, Philadelphia, PA, United States

**Keywords:** working dog, hydration, core body temperature, brain temperature, drinking, ear temperature, exercise recovery, pulse rate

## Abstract

Exercise-related physiological changes were evaluated in hydrated, exercise-conditioned working dogs with free access to tap water (TW) with or without a nutrient-enriched water supplement (NW). Physiological samples and measures were collected before and after work-related field tasks in a warm and moderately humid ambient environment. In a cross-over design study, 12 dogs (age range 8–23 months) were evaluated on 3 separate occasions within each period with exercise bouts up to 30 min, on days −4, 3, and 11. Dogs were offered either *ad libitum* TW or portion-controlled NW daily plus *ad libitum* TW. Prior to and serially after exercise, pulse rate (PR), core (BT_core_) and ear (BT_ear_) temperature were recorded. Urine was collected first thing in the morning, whereas blood samples collected and body weight (BW) recorded pre- and immediately post exercise. Ambient temperature was above 21.7°C (71°F) and relative humidity ranged from 36 to 76%. Activity parameters, AM urine measures, post-exercise percent change of BW, resting PR and resting BT_core_ did not differ between treatment groups on any exercise day. At the completion of exercise, mean BT_core_ for all dogs ranged from 104.8 to 105.6°F. Immediate post-exercise BT_ear_ was always lower compared to BT_core_ and means ranged from 103.3 to 104.0°F. The effect of time was highly significant (*P* < 0.001) for both BT measures with both BT_core_ and BT_ear_ recovering to resting levels by 60 min post exercise. PR and several blood values showed a significant main effect of time. Over the recovery period, dogs in the NW group had lower mean BT_ear_ and PR by 0.6°F and 3.4 bpm, respectively. Daily ingestion of a NW in combination with free access to TW can reduce the post-exercise-related BT_core_ and BT_ear_ hyperthermia, and improve pulse rate recovery following exercise in this population of working dogs undergoing 30 min bout of exercise.

## Introduction

Dogs are natural athletes and perform many work or service-related tasks that involve physical activity. Therefore, the multi-systemic effects of exercise and factors that limit performance are of importance. During exercise, dogs exert energy that leads to heat generation combined with insufficient heat dissipation that results in the natural process of hyperthermia (1–5). Hyperthermia has been documented in dogs in response to exercise at various durations and intensities, and is an important physiological measure of thermoregulation and heat stress risk, but is also a factor limiting performance during physical activity (6–8). In early canine studies, exercise performance could be prolonged by peripheral cooling during exercise leading to a reduced level of hyperthermia, observed by lower rectal (BT_rec_), hypothalamic, and muscle temperatures (4, 5). Recent canine exercise studies have used more sophisticated methods to monitor BT in exercising dogs with minimal interference during or after exercise by recording core body temperature (BT_core_) with ingestible sensors (9–12) and ear temperature (BT_ear_) with non-contact infrared thermography of the tympanic membrane (12, 13) that is representative of brain temperature in people (14, 15), but yet to be confirmed as brain temperature in dogs.

Temperature regulation in dogs is primarily a function of respiratory exchange and associated evaporative heat loss (1). The combination of exercise and evaporative cooling through panting leads to not only hyperthermia (1), but also increased heart rate and panting (7), concomitant increase of respiratory water loss contributing to a transition from a eu-hydrated (true-hydration) state to a hypohydrated (dehydrated) state (16, 17), and many other physiological changes (8, 12). In addition to the effect of thermal regulation on exercise, one of the earliest studies in canine athletes reported that dehydration can negatively impact exercise performance (18), such that dehydrated dogs conserve body water at the expense of higher BT. Reduced or impaired performance is partially related to the effect of dehydration on thermoregulation either prior to exercise and/or becoming dehydrated during exercise. More specifically, dehydrated dogs at rest maintain higher rectal and hypothalamic temperatures (17). Dogs that commence exercise in a dehydrated state develop higher BT_rec_ at the end of a 30 min exercise compared to adequately hydrated dogs (7, 16, 19). In addition, dehydrated dogs have a lower carotid blood flow and lower cardiac output during exercise (7), as well as lower rate of evaporative water loss during 30-min after exercise (16), which will contribute to greater hyperthermia, but will aid in reducing water loss.

Although a variety of canine exercise studies have examined hyperthermia and water loss, they have largely focused on physiological changes while dogs were dehydrated or provided a hypertonic solution (model to increase plasma osmolality and not change plasma volume) compared to “hydrated” dogs with free access to tap water (4, 5, 7, 16, 17, 19). Only a few nutrition studies have examined the use of a water supplement to enhance hydration or reduce the risk of dehydration in dogs on exercise-related dehydration (20) or physiological changes (21). Evaluation of an electrolyte-enriched solution did not demonstrate a benefit to reduce post-exercise BT (21, 22), whereas the use of a glycerol solution reduced exercise-related dehydration (20). No studies appear to have been performed to examine if a nutrient-enriched water supplement (NW) containing glycerol and/or other nutrients might influence exercise-related changes in BT in dogs. By contrast, a great wealth of research has been performed to study the use of water supplements to support the increased water needs, minimize dehydration, and reduce hyperthermia of human athletes (23–26). While estimates of water requirements have been reported for dogs for daily maintenance (27) and exercise (28), no consensus exists for how to define optimal water intake volume in dogs, optimal hydration, or the overall impact of adequate hydration on health (29), which results in the continued reliance on a nutritional recommendation of always having fresh water available for the pet's own desire to ingest water and establish individual eu-hydration (27). More recently, a canine nutrition study evaluated a NW offered as a supplement to *ad libitum* tap water, and demonstrated that non-exercising dogs will have significantly improved daily water intake and hydration (30).

Based on this previous work, this study was designed to evaluate two objectives. First, to evaluate exercise-related physiological changes in hydrated, exercise-conditioned dogs performing in a warm and moderately humid ambient environment that was anticipated to result in sub-clinical to mild dehydration. The physiological measures included for evaluation were exercise hyperthermia of BT_core_ and BT_ear_, pulse rate, venous blood gases, and pre-exercise urine parameters in dogs performing a combination of exercises including search, agility, and retrieving. Second, to determine if a NW that previously was demonstrated to improve hydration in non-exercising dogs (30) would minimize the exercise-related physiological changes that contribute to fatigue, reduced performance or heat stress. Dogs were considered adequately hydrated prior to exercise as they always had free access to tap water or were provided a portion-controlled amount of NW offered daily in combination with free access to tap water. We hypothesized that dogs ingesting the NW in combination with *ad libitum* tap water would demonstrate that exercise-related physiological changes would recover faster or be lower compared to the tap water control group.

## Materials and methods

### Animals, housing, and feeding

As previously described (12), the study protocol (#805342) was approved by the University of Pennsylvania Institutional Animal Care and Use Committee. Twelve exercise-conditioned dogs (8 males and 4 females) of five different breeds; 6 Labrador retrievers, 3 German shepherds, 1 Dutch shepherd, 1 Springer spaniel, and 1 German wirehaired pointer were included. Age of the dogs ranged from 8 to 23 months (median, 16 months). The BW mean was 26.3 kg (± SD 5.8 kg) and ranged from 14.4 to 36.4 kg (31.7 to 80.0 lbs). All dogs used in the exercise trial were required to have overall good general health prior to beginning the trial based on physical examination and were evaluated by a veterinarian at the beginning of each week. All dogs participating in the study had been previously trained to retrieve and perform various agility/search tasks. Exercise conditioning was defined as daily exercise of similar type and duration as used in the exercise protocol for at least 4 weeks preceding the study. Dogs were owned by the University of Pennsylvania and enrolled in the Penn Vet Working Dog Center (PVWDC) training program where each dog trained daily, and lived in a foster home during evenings and weekends. Dogs were individually and temporarily housed in a large (1.5 × 1 × 1.2 m) crate at the PVWDC when not training or exercising during the day. All field exercise and sample collections were conducted on the grounds of the PVWDC.

Daily food intake was monitored throughout the study. All dogs were fed either two or three times daily to maintain an optimal body condition score between 4 and 5 out of 9 (31). Dogs were maintained on commercial dry kibble (Purina Pro Plan Sport All Life Stages Performance 30/20 Formula Dog Food or Pro Plan Focus Sensitive Skin and Stomach Formula Dog Food, Nestlé Purina PetCare Company, St Louis, Mo.) through the duration of the trial. For dogs fed three times daily, the mid-day feeding was delayed until after all post-exercise measurements were taken only on days of sample collection following exercise.

### Experimental design and water supplement treatment

The experimental design was a cross-over and had 2 treatment groups (NW vs. TW) with a total of *N* = 12 dogs per treatment group. For period 1 performed between June 12 and July 1 of 2014, six dogs were randomly assigned to the control group with the remaining assigned to the treatment group. All dogs were switched to receive the alternative treatment group during period 2, which was performed August 7 and August 25, 2014. Both periods consisted of a 4-d baseline phase followed by an 11-d treatment phase.

Starting on day 1 of the treatment phase, test water (NW or TW) was offered in a bowl and was available for 15 min either twice or three times daily depending on whether it was a non-exercise or exercise challenge day, respectively. The volume of test water offered to each dog during the treatment phase was based on each individual dog's calculated water:calorie ratio of 0.25:1 mL/ME kcal, thus 25% of its daily estimated water requirement based on a conservative requirement of 1:1 mL/ME kcal (27). All dogs had *ad libitum* access to TW in a bucket throughout the trial on non-exercise days except for 15 min when the dogs were offered the test water in a food bowl. In addition, dogs were given *ad libitum* access to TW in a bucket before the exercise challenge and after the 15-min period when test water was offered. TW intake from the buckets were not measured.

On non-exercise days, NW or TW was offered twice, in the morning (between 08:00–09:00) and late afternoon (between 14:00–15:00) in a food bowl. Thus, all dogs received a bowl filled with a designated volume of either the NW or TW 2 times for a total of 50% of estimated daily water requirement. On exercise days, all dogs received either the NW or TW 3 times for a total of 75% of estimated daily water requirement; morning, immediately after the 5 min post-exercise body temperature reading was recorded, and late afternoon.

The NW was similar to a previous canine nutrition study in sedentary dogs at rest (30) and was formulated with 94% water, 2.4% whey protein, 1% glycerin, 1% poultry flavor, 0.6% gums, and 1.0% vitamins and minerals. Test water ingestion (NW or TW) was not measured, but observational recording of NW intake based on yes or no assessment. This was based on several reasons. First, on the weekend days, test water offering occurred at the home of the foster pet-owner using a designated container marked with a fill-line to provide a close approximation for the individual dog's specific dose. Therefore, specific measurement of liquid intake in the foster's home was not possible. From Monday through Friday, test water offering occurred at the PVWDC. Second, because the dogs were housed with foster parents before and after training, all other water intake occurrences could not be tracked, which would have included *ad libitum* tap water intake prior to arrival at the PVWDC, which we deemed a possible confounding issue that could influence the ingestion of tap water for either test group prior to be offered the AM test liquid, just risking analysis of erroneous and incomplete AM water intake data prior to exercise. Third, all dogs had *ad libitum* access to TW from a bucket immediately following the 15 min test water offering period and throughout the entire post-exercise and overnight period while at home with the foster, which was also not measured. While not able to be confirmed with this study, our initial water intake study in sedentary dogs (30) demonstrated that offering this daily dose of NW resulted no difference in daily TW ingestion when offered *ad libitum* in a bucket, but does result a significant 36% increase in daily total liquid intake because of ingestion of the NW from a bowl. These sedentary dogs also had significantly diluted urine osmolality after 1 week of NW ingestion. Therefore, we replicated that water administration regimen for this study and incorporated the third test water dose at 5-min post-exercise challenge.

### Exercise regimen

A portion of the exercise field study was previously described (12). Briefly, the study here reports the dogs performing up to a 30-min exercise challenge on 3 separate days over a 15-day period (days −4, 3, and 11). Before every exercise challenge, an initial warm-up included a period of trotting accompanied by active stretches for 5 min. The outdoor exercise challenge was designed to be ≤30 min long; consisting of 5 min of search, followed by 5 min of rest in the shade, then 5 min of agility, followed by 5 min of rest in the shade, and a maximum of 10 min of ball retrieve, and then a cool down period of light trotting/quick walking for 5 min as the dog was returned to the indoor kennel. The start time of each exercise component was documented manually. The search component of the exercise challenge consisted of each dog locating and alerting on 2–3 hidden scent sources (live humans or human remains) in a simulated rubble pile. The agility portion consisted of dogs climbing elevated vertical and horizontal ladders angled 0–45°, walking over unstable surfaces, cavalettis, performing distance exercises (e.g., direction and control), and moving through tunnels, in a controlled pace set by the trainer. Following success in agility or search, dogs were rewarded with tug or ball play. For the rest components dogs were held on leash by a stationary trainer.

Dogs were allowed to sit, stand or walk within the six-foot length of their leash. The ball retrieve component consisted of dogs retrieving a ball or toy. Dogs were allowed up to 15 s between retrieves. Because some dogs did not naturally retrieve, they were trotted on leash in a ball-retrieve fashion until exercise was stopped. Dogs were allotted 15 s between trots. Each exercise portion (search, rest, agility, and ball retrieve) occurred in the same designated location and order for each dog in each trial. The exercise test was stopped if dogs showed signs of curled tongue while panting, seeking shade, or slowing of retrieve. To keep the exercise completion consistent among dogs, a single experienced canine trainer (the PVWDC training manager) determined the end of exercise in each trial (12).

The exercise challenge started at approximately 12:00 p.m. each day for the first dog to allow for moderate external ambient temperature challenge on the five sample collection days for both experimental periods. The order of dogs in the exercise challenge was randomized on each sample collection day, except when medical reasons dictated otherwise. Tracheobronchitis, with no signs of fever or systemic illness, was first recognized in one dog on August 18th. On August 19th, two more dogs were also observed coughing. By August 26th, all but two dogs were observed with a non-productive cough. No difference in performance or motivation was noted by the dog handlers. When controlling for breed, stamina was not influenced if the dog was affected by tracheobronchitis [*p* = 0.67; (12)].

### Sample collection and physiological or biochemical measurements

Exercise day sample collection occurred on day −4 of baseline and days 3 and 11 of treatment phase. All sampling days included body weight, urine collection by free catch in AM prior to exercise, blood sample pre- and immediately post-exercise, locomotor activity from 8 a.m. to 4 p.m., and six body temperature (BT) and pulse-rate (PR) recordings.

Manual pulse rate was obtained from the femoral artery. BT was measured by two methods to assess both brain and core body temperature (BT_core_). Temperature measured from the tympanic membrane of the left ear (BT_ear_) was collected using an instant ear thermometer device [Pet-Temp® Instant Infrared Ear Thermometer for Home Use, Model PT-300, Advanced Monitors Corporation, San Diego, California; (13)]. BT_core_ was measured using a telemetric core temperature monitor (CorTemp® Indigestible Core Body Temperature Sensor, HQInc., Palmetto, Florida) to obtain temperature within the gastrointestinal tract (12). New CorTemp sensors were swallowed by the dogs the morning of each exercise challenge day. BT_core_ data measured by the CorTemp sensor were collected wirelessly by the CorTemp Data Recorder (HQInc., Palmetto, Florida).

Two samples of venous blood (~3 mL; jugular, cephalic, or saphenous) were collected on each exercise challenge day in the morning between 07:00 and 09:00 a.m. (pre-exercise) and immediately (0-min) post-exercise before the cool down period. Blood samples were analyzed immediately for blood pH, gases (PvCO_2_, HCO_3_, TCO_2_, PvO2, SvO_2_), electrolytes (iCa, Na, K), base excess in extracellular fluid compartment (BEecf), glucose, hemoglobin, and hematocrit using point of care i-STAT CG8+ cartridge (Abaxis, Union City, California) with a VetScan i-STAT 1 Handheld Analyzer (Abaxis, Union City, California). Blood lactate was measured using point of care analysis (Lactate Scout+, EKF Diagnostics GmbH, Magdeburg, Germany). Remaining blood was transferred to blood tubes (BD Vacutainer SST tubes, Becton, Dickinson and Company, Franklin Lakes, NJ) and allowed to clot for 10 min at room temperature. Serum was collected after centrifugation of clotted blood samples and stored at −80°C until aliquoted samples were analyzed for osmolality using an osmometer (Vapro 5520 Vapor Pressure Osmometer, Wescor Inc., Logan, Utah) and total protein and urea nitrogen by means of an automated biochemical assay system (Cobas, model c311, Roche Diagnostics, Indianapolis, Indiana) in accordance with the manufacturer's instructions.

Urine samples of free-catch voided urine were collected when the dogs were walked and a collection pan on the end of a pole was used to catch the urine as the dog voided voluntarily. Urine samples were stored at −80°C until analyzed for specific gravity with a refractometer (HSK-VET, Veterinary refractometer, Heska, Loveland, Colorado). pH (pH 11 Meter, Oakton Instruments, Vernon Hills, Illinois), Na^+^, total protein, urea nitrogen and creatinine by means of an automated biochemical assay system (Cobas, model c311, Roche Diagnostics, Indianapolis, Indiana).

### Locomotor activity monitoring

Locomotor activity was measured using an omnidirectional accelerometer device (version 3.1, Actical®, Respironics, Koninklijke Philips Electronics, Bend, Oregon) placed inside a specially designed case and attached to the dog's collar between 8 and 10 a.m., and taken off after final post-exercise 60-min measurements. The activity device was set to record activity counts with a 1-min epoch. The total activity data encompassed the warm up (5-min period of trotting/stretching before start of search exercise), exercise period, and the cool down (5-min period of trotting/stretching after end of ball retrieve exercise). The activity data files were batch processed using the Actical software (version 3.1, Actical®, Respironics, Koninklijke Philips Electronics, Bend, Oregon). All epoch data was consolidated into a single Microsoft Excel file for alignment of activity count time-stamp with the manually recorded time of exercise.

### Statistics

To examine differences between treatment groups, two primary analytical techniques were used; linear mixed effects models and paired samples *t*-tests. When the same animal was measured multiple times in each condition (e.g., pre and post exercise), the linear mixed effect model was used to account for the repeated nature of the data using the lmer package (32). Unless otherwise specified, time, treatment, and their interaction were entered as fixed effects and dog ID was entered as the random effect. The Satterthwaite equation was used to estimate the degrees of freedom. When there was only one measurement per dog in each condition, paired sample *t*-tests were conducted. All analyses were conducted using R [(33), version 3.4.1. Accessed 11-2017 through 01-2018] and were considered to be significant at α = 0.05.

## Results

### Ambient environmental conditions, exercise-related locomotor activity, body weight, and pre-exercise urine measures

Ambient temperature and humidity on exercise days were generally similar and temperature was always above 21.7°C (71°F). Mean [min-max range] ambient temperature on day −4, 3, and 11 for both experimental periods were 26.2°C (79.1°F) [21.7–30°C], 28.4°C (83.2°F) [22.2–34.4°C], and 28.0°C (82.4°F) [25.0–29.4°C], respectively. Relative humidity ranged from a low of 36–39% to a high of 67–76% across all exercise days.

Locomotor activity counts were recorded during the 30-min bout of exercise to assess exercise-related activity as a potential factor influencing body temperature between treatment groups and between different exercise days. Exercise duration and intensity were assessed by recording locomotor activity counts and duration of activity, which were reported as total activity (sum of warm up before exercise, during exercise, and cool down after exercise) and exercise-only activity (Table 1). The main effects of treatment, time, as well as the 2-way interaction from the linear mixed effect model, were not significant for any activity parameter.

**Table 1:** Mean (±SE) locomotor activity counts, activity duration, and morning (pre-exercise) urine measures in exercise-conditioned dogs (*N* = 12) performing a 30-min exercise challenge on days −4, 3, and 11 when offered TW or a NW in addition to *ad libitum* access to TW in a bucket.

				***p***-values*		
**Measures and treatment groups**	**Day −4**	**Day 3**	**Day 11**	**Day**	**Trt**	**Trt × Day**
**TOTAL ACTIVITY DURATION, MIN**						
TW	37.5 ± 1.8	40.7 ± 0.9	41.1 ± 0.8	0.10	0.44	0.61
NW	39.7 ± 1.4	40.4 ± 1.0	41.5 ± 0.9			
**TOTAL EXERCISE DURATION, MIN**						
TW	26.8 ± 0.8	27.1 ± 0.8	27.2 ± 0.6	0.95	0.56	0.93
NW	27.4 ± 0.7	27.2 ± 0.7	27.5 ± 0.6			
**TOTAL ACTIVITY COUNTS, ARBITRARY UNITS**						
TW	73.899 ± 4.981	80.227 ± 5.676	73.147 ± 7.379	0.50	0.27	0.69
NW	71.611 ± 2.373	72.334 ± 4.475	70.566 ± 3.066			
**TOTAL EXERCISE COUNTS, ARBITRARY UNITS**						
TW	53.386 ± 3.887	53.712 ± 4.768	48.753 ± 2.850	0.76	0.32	0.74
NW	47.901 ± 2.183	49.081 ± 3.597	49.185 ± 2.856			
**CREATININE, mg/dL**						
TW	206 ± 15	239 ± 16	211 ± 13	0.59	0.64	0.40
NW	212 ± 21	209 ± 16	217 ± 16			
**pH**						
TW	6.3 ± 0.3	6.3 ± 0.2	6.4 ± 0.3	0.16	0.54	0.72
NW	6.2 ± 0.2	6.3 ± 0.2	6.5 ± 0.2			
**USG, g/mL**						
TW	1.044 ± 0.004	1.045 ± 0.003	1.042 ± 0.004	0.30	0.44	0.69
NW	1.044 ± 0.002	1.043 ± 0.003	1.048 ± 0.003			
**SODIUM, mmol/L**						
TW	122 ± 17	139 ± 20	182 ± 25	0.01	0.71	0.79
NW	142 ± 23	134 ± 18	185 ± 20			
**TOTAL PROTEIN, mg/dL**						
TW	30 ± 6	31 ± 5	29 ± 4	0.88	0.44	0.68
NW	29 ± 5	28 ± 4	29 ± 5			
**UREA NITROGEN, mg/dL**						
TW	3.362 ± 398	3.098 ± 293	2.774 ± 303	0.51	0.44	0.16
NW	2.949 ± 118	2.749 ± 238	3.119 ± 341			

Dogs of the TW (N = 12) and NW (N = 12) groups received TW in a bucket and dry kibble during the week before the treatment period (i.e., baseline; days −7 to −1). No changes were made to the food and water regimen for dogs of the TW group from days 0 through 11, except for the addition of TW offered in a bowl at designated times similar to the NW group. On days 0 through 11, dogs of the NW group received NW in a bowl and still had ad libitum TW in a bucket.

* *P-values were generated from a linear mixed model*.

Although test water intake volume pre or post-exercise was not measured, all dogs offered the NW always ingested the entire volume while at rest or post-exercise, whereas dogs offered the TW when at rest were observed to drink either nothing or varying amounts within the 15-min liquid offer period. Post-exercise, all dogs offered TW in a bowl ingested some or all TW volume offered during the 15 min period. Body weight (BW) was measured before and immediately after exercise on all 3 exercise challenge days to assess acute body water loss and estimate post-exercise dehydration. The percent change of BW post-exercise was analyzed by paired samples *t*-test to examine the differences between NW and TW treatment groups on each exercise challenge day. Percent change of BW did not differ (*P* > 0.17) between treatment groups for any exercise day. Although not different between treatment groups, an acute mild loss of body water occurred after each 30 min exercise. Specifically, the mean (± SE) % loss of BW for all dogs was 1.3% (±0.4%), 1.6% (±1.1%), and 1.5% (±0.6%) for days −4, 3, and 11, respectively.

Free-catch urine samples were collected in the morning and representative of pre-exercise effect. For all urine parameters measured (Table 1), no main effects for day, treatment or the two-way interaction from the linear mixed model were observed, with the exception of a day effect for sodium, in which both groups had a higher concentration on day 11.

### Body temperature and pulse rate measures

**Figure 1:**
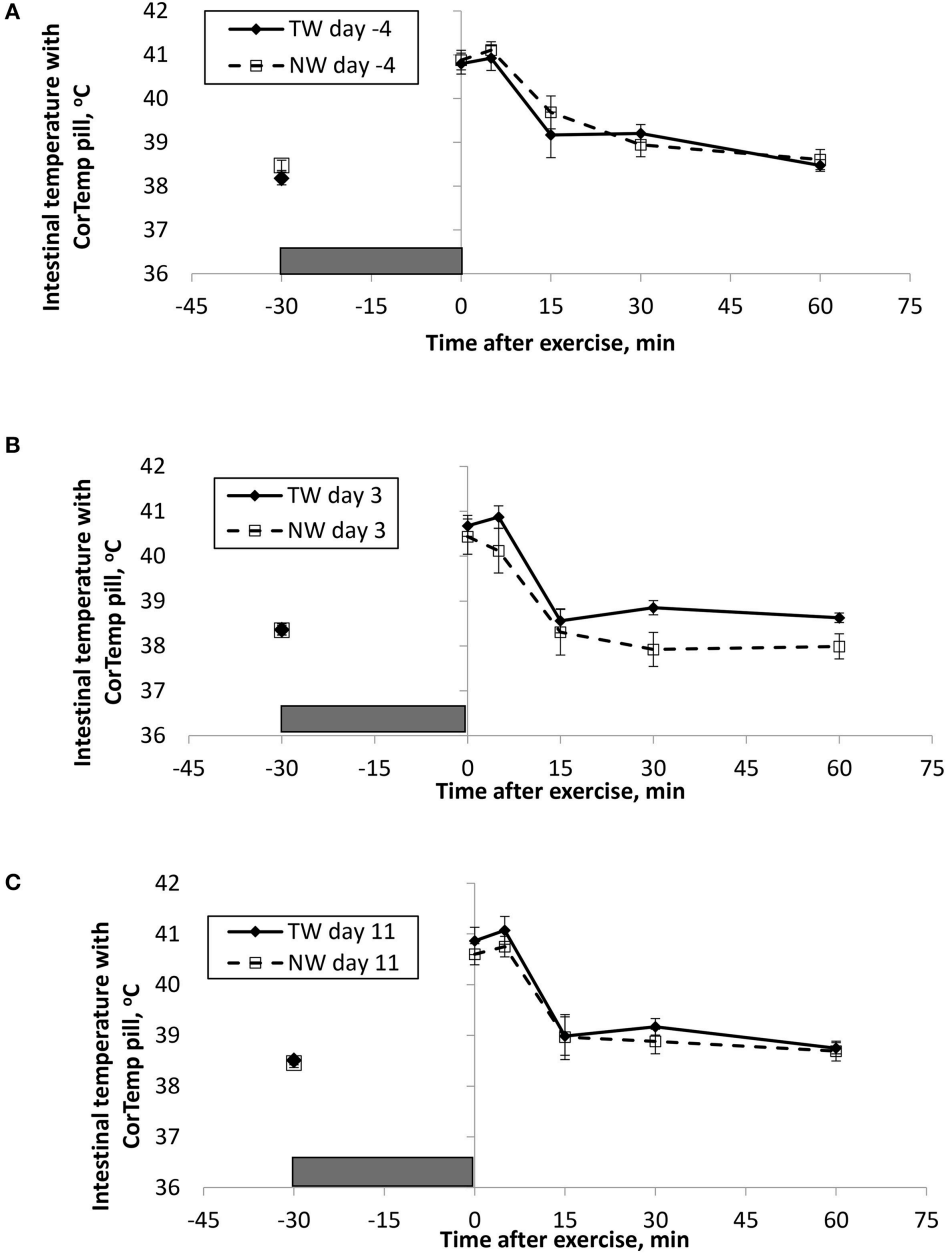
Mean (±SE) gastro-intestinal body temperature recorded with the CorTemp pill 30 min before and multiple times after a 30 min exercise on days **(A)** −4, **(B)** 3, and **(C)** 11 of the treatment phase when dogs had free access to tap water (TW) or portion-controlled nutrient-water (NW) and free access to TW. The gray box represents the 30-min exercise period. Dogs were assigned to TW (*n* = 12) or NW (12) groups; all dogs received TW for drinking during the week before the treatment phase (i.e., day −4). No changes were made to the food and water regimen for dogs of the TW group during the treatment phase. Dogs of the NW group received NW twice daily from days 0 through 11, and were offered the NW immediately after the 5-min post-exercise measures were recorded. Student's *t*-test was performed to compare treatment groups for baseline −30 min pre-exercise temperature. A linear mixed model was performed to analyze the main effects of treatment and post-exercise time, and 2-way interaction.

**Figure 2:**
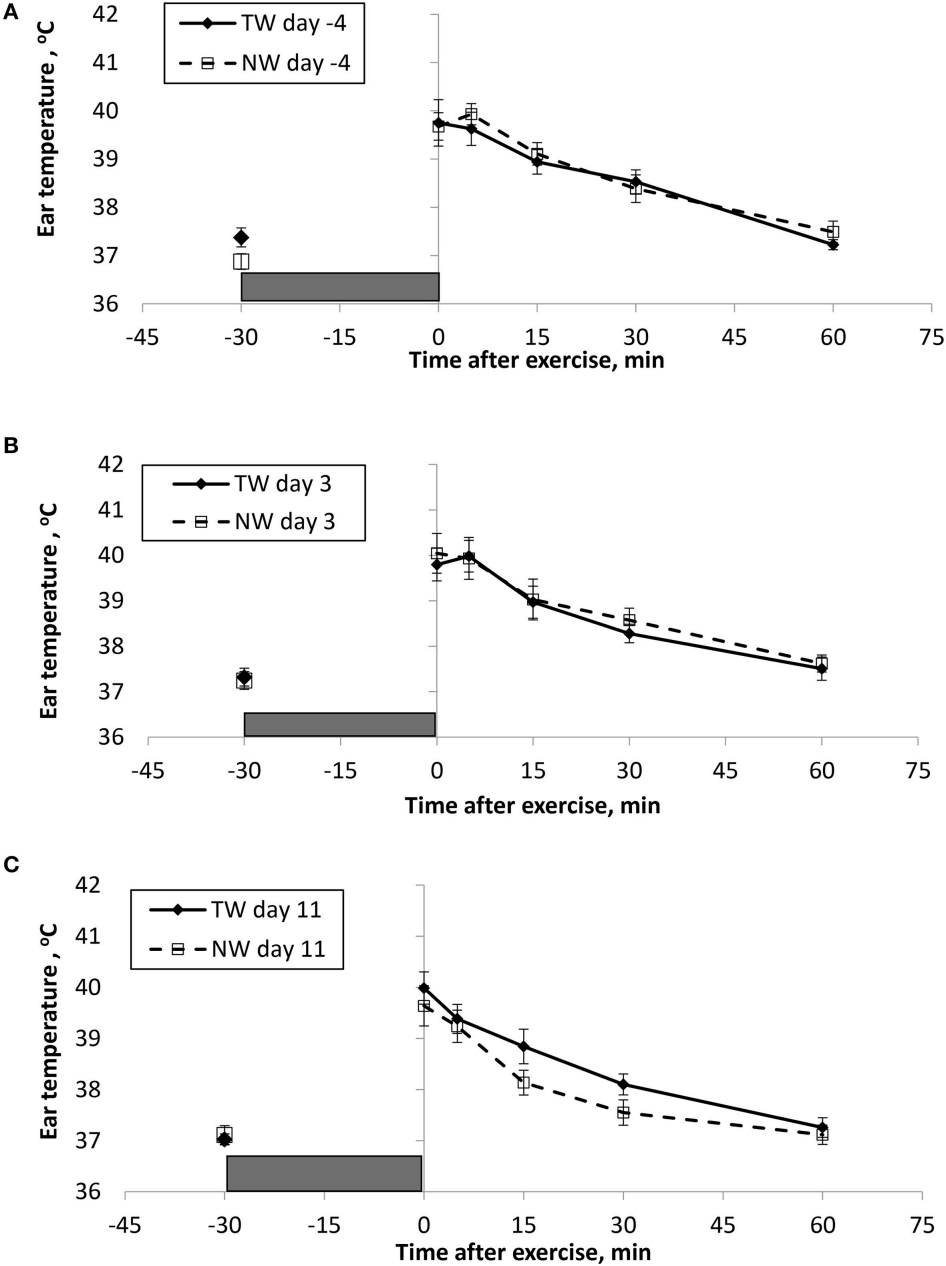
Mean (±SE) ear temperature recorded 30 min before and multiple times after a 30 min exercise on days **(A)** −4, **(B)** 3, and **(C)** 11 of the treatment phase when dogs had free access to tap water (TW) or portion-controlled nutrient-water (NW) and free access to TW. The gray box represents the 30-min exercise period. Dogs were assigned to TW (*n* = 12) or NW (12) groups; all dogs received TW for drinking during the week before the treatment phase (i.e., day −4). No changes were made to the food and water regimen for dogs of the TW group during the treatment phase. Dogs of the NW group received NW twice daily from days 0 through 11, and were offered the NW immediately after the 5-min post-exercise measures were recorded. Student's *t*-test was performed to compare treatment groups for baseline −30 min pre-exercise temperature. A linear mixed model was performed to analyze the main effects of treatment and post-exercise time, and 2-way interaction.

**Figure 3:**
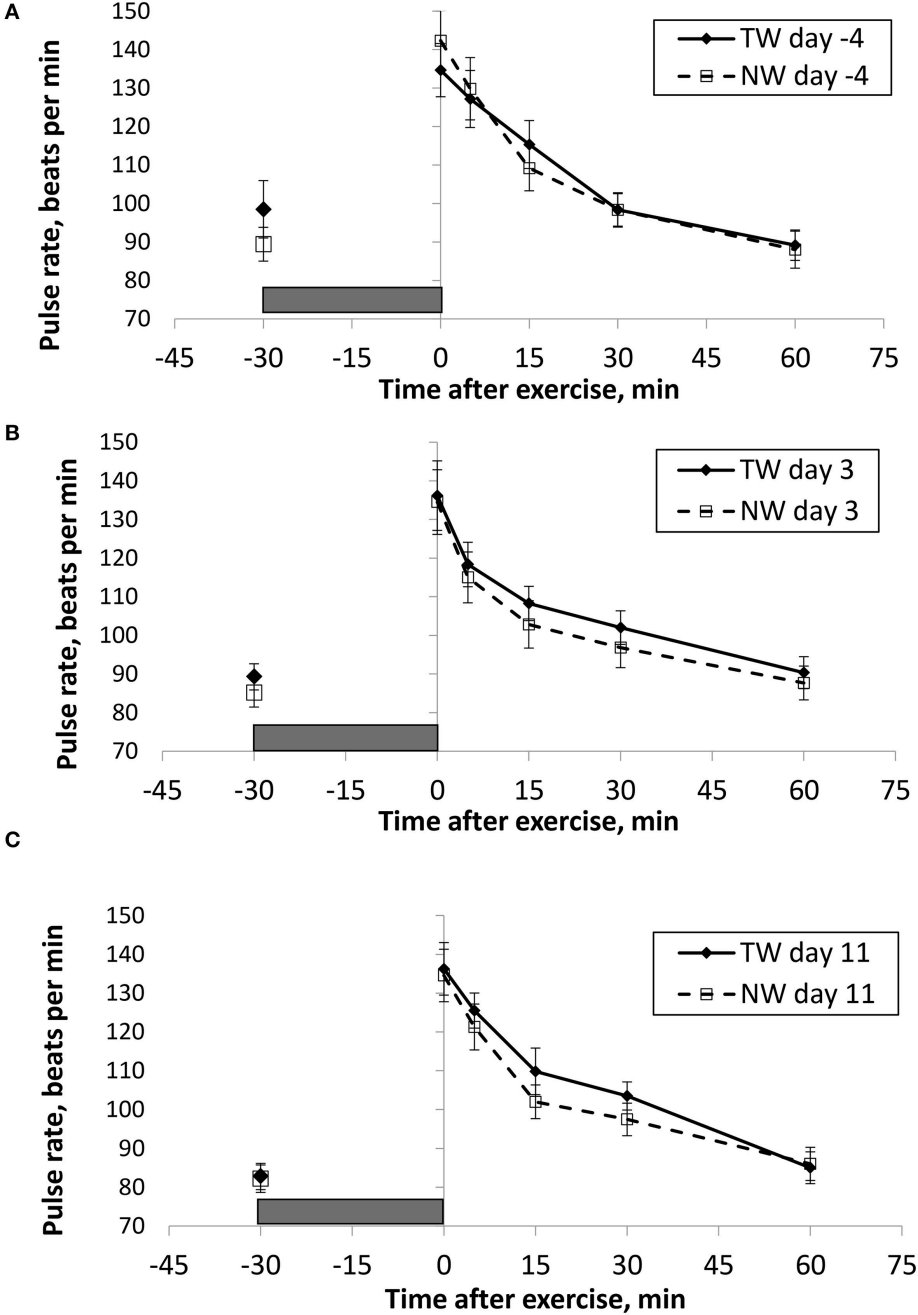
Mean (±SE) manual pulse rate (beats/min) recorded 30 min before and multiple times after a 30 min exercise on days **(A)** −4, **(B)** 3, and **(C)** 11 of the treatment phase when dogs had free access to tap water (TW) or portion-controlled nutrient-water (NW) and free access to TW. The gray box represents the 30-min exercise period. Dogs were assigned to TW (*n* = 12) or NW (12) groups; all dogs received TW for drinking during the week before the treatment phase (i.e., day −4). No changes were made to the food and water regimen for dogs of the TW group during the treatment phase. Dogs of the NW group received NW twice daily from days 0 through 11, and were offered the NW immediately after the 5-min post-exercise measures were recorded. Student's *t*-test was performed to compare treatment groups for baseline −30 min pre-exercise temperature. A linear mixed model was performed to analyze the main effects of treatment and post-exercise time, and 2-way interaction.

BT (Figures 1, 2) and PR (Figure 3) were measured before and at various times after a 30 min bout of exercise to characterize the effect of exercise on changes in BT between the treatment groups on multiple days during the study. To assess the resting (pre-exercise) BT_ear_ and BT_core_ between treatment groups, a paired samples *t*-test was performed using the −30 min data recorded on each exercise day. Resting BT_core_ did not differ (*P* > 0.17) between treatment groups before the start of exercise on any exercise day. BT_ear_ differed on day −4, in which the TW group mean was 1.1°F higher (*P* = 0.01) than the NW group. Resting PR did not differ (*P* > 0.32) between treatment groups on any exercise day.

At the completion of exercise, mean BT_core_ for both groups and over the 3 exercise days ranged from 104.8 to 105.6°F, and on each day the immediate (0-min) post-exercise group means varied by 0.2–0.4°F between groups. Immediate post-exercise BT_ear_ was always lower compared to BT_core_ and group means ranged from 103.3 to 104.0°F. Immediate post-exercise means between treatment groups for BT_ear_ varied by < 0.1°F, except for on day 11 when means differed by 0.7°F (TW, 104.0°F vs. NW, 103.3°F).

BT and PR were analyzed for the main effects of time, treatment, and their interaction using a linear mixed model to assess the effect of post-exercise recovery (all data recorded from 0-min through 60-min post exercise) and the influence of test liquid ingestion after exercise. On day −4 when all dogs were ingesting TW, BT_core_ and BT_ear_ were not significant for the main effect of treatment (*P* = 0.45 and *P* = 0.59, respectively), or for the time-by-treatment interaction (*P* = 0.70 and *P* = 0.91, respectively). The effect of time was highly significant (*P* < 0.001) for both BT measures with both BT_core_ and BT_ear_ recovering to resting levels by 60 min post exercise. PR also resulted in a significant main effect of time (*P* < 0.001), but not for treatment (*P* = 0.84) or time-by-treatment interaction (*P* = 0.66).

In contrast to day-4, BT_core_ on day 3 had both a significant effect of time (*P* < 0.001) and treatment (*P* = 0.002) in the linear mixed model, in which the NW group (38.9°C) had a 0.6°C (1°F) lower overall BT_core_ compared to the TW group (39.5°C). BT_ear_ and PR did not result in a significant main effect of treatment (*P* = 0.88 and *P* = 0.32, respectively) or time-by-treatment interaction (*P* = 0.99 and *P* = 1.0, respectively). Similar to day −4, both BT_ear_ and PR declined over time after exercise (*P* < 0.001).

On day 11, BT_core_ did not differ between treatments (*P* = 0.19) in the linear mixed model. Interestingly, both BT_ear_ (*P* = 0.003) and PR (*P* = 0.03) resulted in a significant main effect of treatment, in which dogs in the NW group had an overall mean BT_ear_ and PR that was lower by 0.6°F and 3.4 bpm, respectively, over the entire 60-min recovery period. It is notable that the greatest quantitative difference between treatment groups was observed for the same time points post-exercise (15- and 30-min) for both BT_ear_ and PR. The main effect of time was significant for both BT measures and PR (*P* < 0.001), and 2-way interactions were not different (*P* > 0.64).

Finally, there was a significant (*p* < 0.03), positive linear correlation between BT_core_ and PR (*r* = 0.71;) when relating measures obtained at immediate post-exercise time, as well as for BT_ear_ and PR (*r* = 0.46).

### Blood measures

The serum analyte concentration for pre- and post-exercise data were summarized (Table 2). Many blood variables differed (*P* < 0.001) with the main effect of time to assess pre- vs. post-exercise, in which several variables were lower immediately after exercise (BE_ecf_, pCO_2_, HCO_3_, iCal, TCO_2_), whereas many others increased after exercise (hemoglobin, glucose, pO_2_, pH, lactate, hematocrit, SO_2_). Serum osmolality, potassium, sodium, total protein, and urea nitrogen concentrations did not differ after exercise compared to pre-exercise. No 2-way or 3-way interactions, nor the main effect of day (data not shown) or treatment were statistically significant for any variable.

**Table 2:** Mean (±SE) blood measures pre- and immediately post-exercise in exercise-conditioned dogs (*N* = 12) performing a 30-min exercise challenge on days −4, 3, and 11 of treatment phase when offered TW or a nutrient-enriched water in addition to *ad libitum* access to TW in a bucket.

	**Independent variables for time**		***p***-values*						
**Measures**	**Pre-exercise**	**Post-exercise**	**Time**	**Trt**	**Day**	**Time × day**	**Trt × time**	**Trt × day**	**Time × day × trt**
BE_ecf_, mmHg	−3.64 ± 0.26 (−7 – 1)	−4.58 ± 0.27 (−10 – −1)	<0.001	0.09	0.86	0.66	0.19	0.98	0.85
pCO_2_, mmHg	37.6 ± 0.5 (27.2–50.4)	20.5 ±0.8 (8.70 – 38.8)	<0.001	0.88	0.84	0.97	0.64	0.85	0.96
Glucose, mmol/L	88.9 ± 1.1 (70.0–110.0)	99.9 ± 1.1 (82.0–123.0)	<0.001	0.37	0.31	0.79	0.99	0.96	0.95
Hemoglobin, g/dL	14.6 ± 0.2 (11.9–18.7)	15.5 ± 0.1 (13.9–18.0)	<0.001	0.38	0.25	0.61	0.45	0.95	0.71
HCO_3_, mmHg	21.5 ± 0.3 (13.3–26.2)	17.6 ± 0.3 (12.9–22.9)	<0.001	0.15	0.97	0.96	0.97	0.51	0.93
iCal, mmol/L	1.45 ± 0.04 (1.27–4.50)	1.27 ± 0.01 (1.07–1.60)	<0.001	0.36	0.28	0.31	0.46	0.35	0.50
S_osm_, mOsm/kg	307 ± 1 (287–329)	308 ± 1 (290–332)	0.75	0.99	0.27	0.30	0.27	0.28	0.94
pO_2_, mmHg	45.9 ± 2.3 (23–134)	72.9 ± 4.0 (33–181)	<0.001	0.57	0.15	0.22	0.92	0.92	0.97
pH	7.37 ± 0.01 (7.31–7.44)	7.56 ± 0.1 (7.37–7.83)	<0.001	0.47	0.94	0.83	0.43	0.92	0.98
Potassium, mmol/L	4.30 ± 0.03 (3.40–5.20)	4.34 ± 0.03 (3.80–5.00)	0.35	0.82	0.69	0.61	0.69	0.55	0.21
Lactate, mmol/L	1.16 ± 0.04 (0.6–2.2)	1.45 ± 0.07 (0.6–4.1)	<0.001	0.41	0.44	0.97	0.93	0.67	0.99
Hematocrit, %PCV	43.1 ± 0.5 (35–55)	45.7 ± 0.4 (41–53)	<0.001	0.40	0.24	0.59	0.41	0.95	0.72
SO_2_, %	74.2 ± 1.7 (40–99)	92.9 ± 0.8 (64–100)	<0.001	0.64	0.17	0.48	0.80	0.83	0.80
Sodium, mmol/L	145 ± 1 (140–149)	145 ± 1 (142–148)	0.38	0.77	0.03	0.93	0.85	0.18	0.67
TCO_2_, mmHg	22.8 ± 0.3 (19–28)	18.2 ± 0.3 (13–24)	<0.001	0.23	0.75	0.93	0.54	0.70	0.82
Total protein, g/dL	5.78 ± 0.05 (4.90–6.90)	5.81 ± 0.05 (4.90–6.60)	0.56	0.71	0.10	0.98	0.51	0.79	0.30
Urea nitrogen, mg/dL	23.2 ± 0.6 (12.9–48.9)	23.0 ± 0.5 (13.8–36.9)	0.97	0.88	0.07	0.22	0.65	0.09	0.86

Dogs of the TW (N = 12) and NW (N = 12) groups received TW in a bucket and dry kibble during the week before the treatment period (i.e., baseline; days −7 to −1). No changes were made to the food and water regimen for dogs of the TW group from days 0 through 11, except for the addition of TW offered in a bowl at designated times similar to the NW group. On days 0 through 11, dogs of the NW group received NW in a bowl and still had ad libitum TW in a bucket.

* *P-values were generated from a linear mixed model.Minimum–maximum range in brackets*.

## Discussion

This data further elaborates on our initial report that exercise-conditioned working dogs that performed up to 30-min of field exercise consisting of rubble search, agility, and retrieving in warm ambient temperature experienced a significant elevation in BT and changes in serum chemistries and blood gases (12). Exercise-related hyperthermia and subsequent BT and PR recovery response following exercise were documented to further characterize this exercise model that results in sub-clinical (<5% BW loss) dehydration. The primary set of unique findings reported in this study is that daily ingestion of a portion-controlled volume of a NW in combination with free access to tap water can reduce the post-exercise-related BT_core_ and BT_ear_ hyperthermia, as well as improve pulse rate recovery following exercise in this population of working dogs undergoing 30 min bout of exercise.

In general, our post-exercise BT_core_ data generated with the thermistor pill are consistent with previous exercise studies that reported elevated rectal temperature to approximately 40–41°C (104–105.8°F) in dogs that performed 25–30 min of treadmill running (16, 34–36) or 30 min of vigorous play exercise with retrieving (13). Slightly different from previous studies with dogs on treadmills was that our immediate post-exercise femoral artery PR of approximately 140 bpm was lower than that observed by measuring ECG heart rates of approximately 170–190 bpm (34) or femoral PR of 160 bpm (35). Although these studies (12, 16, 34–36) evaluated exercise-related hyperthermia, none reported total body water, hydration status, or post-exercise BW loss as a measure of acute body water loss. Much earlier work with dogs that performed treadmill exercise documented BT changes related to moderate dehydration, but dehydration occurred prior to exercise and was moderate to borderline severe ranging from 9 to 10% loss of BW (7, 16). However, data with hydrated working dogs that become sub-clinically (<5% loss of BW) to mildly dehydrated (5–7% loss of BW) resulting from routine field exercise is lacking.

Although subclinical dehydration is seemingly inconsequential, only recently has it become apparent that mild hypohydration (<2%) can have both cognitive and exercise performance implications in people. Studies with young adults (men and women) and children indicated that dehydration of < 2% loss of BW resulted in impaired cognitive performance and mood (37–39), and dehydrated cyclists with as little as 1% loss of BW had decreased exercise performance (40). To date, this type of data does not exist for working dogs undergoing prolonged (non-sprint) exercise, but one study with racing grayhounds has reported that sub-clinical dehydration (~2.5% BW loss) before the start of the race resulted in some increased likelihood of improved race performance (41). While this improved sprint performance and type of exercise is important for grayhound racing in which dogs will experience little to no exercise-related water loss, it is quite different from working dogs performing from 30 min to several hours of exercise. Thus, understanding mild shifts in dehydration and evaluating strategies to minimize these changes may extend stamina, alertness, and/or performance, as well as reduce the risk of heat stress and progression of dehydration with prolonged exercise.

On all 3 exercise challenge days during both experimental periods, ambient environment temperature was within or slightly above thermoneutral zone of 20–30°C (68–86°F); (28). Exercise duration and intensity performed in this study were similar between exercise days and between treatment groups, and resulted in primarily subclinical dehydration (<5% loss of BW) on all 3 exercise challenge days for most dogs, but some notable exceptions existed on days 3 and 11.

Across the study, all dogs experienced approximately 2.5–3.0°C (4–5°F) increase in body temperature at the end of exercise, and post-exercise BT_core_ was always higher compared to BT_ear_, which is consistent with previous observations in Beagles and Labrador Retrievers (13). Because BT_core_ was measured with the use of a thermistor pill and is representative of gastrointestinal temperature that is not measured at the rectum, it was not surprising that we observed a significant cooling in BT_core_ at the 15-min post-exercise time on every exercise day for all dogs. Presumably this is a result of the pill being cooled in the stomach from liquid ingested in the intervening period following the 5-min post-exercise measurement, and not as a result of an actual drop in BT_core_. More importantly, this study uniquely reports that overall BT_core_ (mean of all post exercise measures) was significantly lower by 0.6°C (1°F) in dogs that ingested the NW compared to dogs drinking only TW. Although the intestinal heat load was lower in the NW group, this did not result in a lower BT_ear_ or PR, and the effect was not replicated on day 11.

Interestingly on day 11, this study uniquely reports that dogs in the NW group demonstrated a significantly lower BT_ear_ after exercise, which also corresponded with lower PR, thus improved recovery following exercise. Past research in people has reported that tympanic membrane and hypothalamus share a blood supply from the carotid arteries (14, 15) and an ear temperature reading is reflective of heat from both tympanic membrane and ear canal (42). Therefore, BT_ear_ reported in sedentary and exercising dogs may also be a surrogate of brain temperature, but needs to be confirmed. BT_ear_ after exercise also appears to be a reasonable alternative to assess core body temperature fluctuations. Early work in dogs has demonstrated that direct elevation of temperature within the anterior hypothalamic regions of the brain resulted in an increase in panting, as well as a subsequent increase in heart rate, cardiac output, and arterial blood pressure, followed by a reversal of the effect with cooler brain temperature (43, 44). Hypothalamic temperature can also be lowered during exercise through cooling of the body, which has also been shown to reduce both heart rate and panting rate in dogs during exercise (4). Thus, dogs drinking the NW experience a benefit of having a lower BT_ear_ after exercise, possibly a reflection of brain temperature, that ultimately is related to reduced heart rate, and presumably panting rate recovery. Importantly, this being observed while at a level of water intake that appears to result in the NW dogs being similarly hydrated compared to dogs drinking TW. In addition, although post-exercise dehydration was similar between groups, NW group dogs were less likely to become mildly dehydrated vs. only sub-clinically dehydrated, however more observations and additional research are needed to further explore this preliminary evidence.

Although exercise-related BW loss did not differ between treatment groups, greater variability was observed on day 3 and 11 of the treatment period for the TW group. Examination of the dataset variability revealed that only 1 dog in the NW group on day 11 would be classified as being mildly dehydrated (5.1% BW loss), whereas in the TW group there were 5 observations of mild dehydration following exercise that ranged from 5.2 to 8.9% loss of BW. Three of these observations occurred on day 3 and 2 occurred on day 11, with a single dog becoming mildly dehydrated on both day 3 and 11, which was not observed when this dog ingested the NW.

In addition to the more traditional estimate of acute dehydration through tracking BW loss, the evaluation of morning concentration of urine determined by osmolality or specific gravity has been recently evaluated and generally associated with overall hydration and daily water intake (29, 30). Specifically, a significant inverse curvilinear relationship has been characterized and reported between USG and total daily water intake (mL/kg BW) in healthy non-exercising dogs, in which greater water intake was associated with more dilute USG, and a presumption of overall improved hydration based on additional reported data (30). Based on this previous work, we hypothesized that the exercising dogs ingesting the NW in this current study would have a greater total water intake and more dilute urine, thus be better hydrated before the start of exercise on day 11 and possibly day 3. However, no difference was observed in morning, pre-exercise USG between either treatment groups or between exercise days compared to baseline. This could be related to urine samples being obtained within a shorter treatment phase (days 3 and 11) compared to the previous study (days 14, 30, and 42) in which a significant dilution of USG was observed.

It is also worth noting that a critical experimental design limitation in the current study was that the dogs involved in the trial were housed in foster homes in the evenings and weekends when not training at the PVWDC, thus it was not feasible to obtain daily total water intake data.

Based on the inverse curvilinear model reported by Zanghi and Gardner (30), one might predict that the dogs used in this current study with a USG ranging from 1.042 to 1.048 g/mL would have been ingesting approximately 35–50 mL/kg BW. This is well below the water intake observed with non-exercise dogs eating a generally similar food that had a much more dilute USG. This also suggests that the Shepherds and Labradors involved in the current study may benefit from not only an increase in total tap water intake, but likely also a greater daily portion of NW to improve hydration and total water intake. This also indicates that even dogs with *ad libitum* access to tap water may not be optimally hydrated, confirming the need for alternative hydration strategies.

Finally, with regards to the specific nutrients in the NW and why dogs in the NW group would have experienced lower post-exercise BT and PR in the absence of any hydration improvement, some prior evidence in dogs and people exists that was initially used to conceive of the NW formulation. Specifically, there is a considerable amount of existing literature in the human nutrition and exercise field associated with organic osmolytes, rehydration, hydration status, and/or thermoregulation. Some of this research has been recently summarized in several review articles (45–47). Among the various organic osmolytes, glycerol has received significant scientific attention while being studied as a water supplement-nutrient for hyperhydration in people, as it has been shown to support improved water retention during post-exercise rehydration (48) and improved thermoregulation (49, 50), as well as demonstrated preliminary evidence of hyperhydration in exercising dogs (20). In addition, other nutrition studies have indicated that amino acids in the form of small peptides (51) or whole protein sources from milk (46) can help offset hydration stress using an exercise model in people. Furthermore, earlier research demonstrated that glutamine supplementation increased electrolyte and water absorption in animal and human models of intestinal infection (52–54). This suggests that the protein sources with various compositions of amino acids in free, peptide, and intake form may provide similar benefits to promoting water absorption and retention.

In conclusion, the putative benefits of a NW containing glycerol and amino acids to minimize dehydration in dogs following exercise was inconclusive within the current experimental design, although some suggestion of decreased severity of water loss in individual dogs was observed that warrants further investigation. This study does demonstrate that the dogs drinking the NW experienced reduced post-exercise BT and PR changes and demonstrates that a water supplement appears to be a viable method for supporting body temperature and heart rate recovery following exercise in hardworking dogs. Because elevated BT and PR contribute to fatigue, reduced performance or heat stress, it is plausible that these factors may be improved, but further investigation is necessary to demonstrate these putative effects. The nutrient composition of this NW supplement ingested twice daily based on an estimated 50% of the dogs' daily water requirement on resting days and 75% on exercise days appears to provide these working dogs with improved thermoregulation that is consistent with exercise studies in people also ingesting a glycerol-enriched solution. Further work with higher volumes of the NW is justified and should elucidate a better understanding for improved liquid administration recommendations that might achieve both enhanced hydration and improved thermoregulation. Ultimately, it will be necessary to determine if exercise performance or working ability is improved or prolonged with any hydration strategy.

## Author contributions

All authors listed have made a substantial, direct and intellectual contribution to the work, and approved it for publication.

### Conflict of interest statement

BZ is employed within the R&D department of Nestlé Purina Pet Care and conducts nutrition research for the potential use in future commercial applications and products.

The remaining authors declare that the research was conducted in the absence of any commercial or financial relationships that could be construed as a potential conflict of interest.

## Abbreviations

AF: as fed
BCS: body condition score
BT: body temperature
BT_core_: core body temperature
BT_ear_: ear temperature of tympanic membrane
BT_rec_: rectal body temperature
BW: body weight
ICP-OES: inductively coupled plasma optical emission spectroscopy
NW: nutrient-enriched water
TW: tap water
U_osmo_: urine osmolality
USG: urine specific gravity.
